# Clinical Characteristics and Outcomes Among Adults Hospitalized with Laboratory-Confirmed SARS-CoV-2 Infection During Periods of B.1.617.2 (Delta) and B.1.1.529 (Omicron) Variant Predominance — One Hospital, California, July 15–September 23, 2021, and December 21, 2021–January 27, 2022

**DOI:** 10.15585/mmwr.mm7106e2

**Published:** 2022-02-11

**Authors:** Matthew E Modes, Michael P. Directo, Michael Melgar, Lily R. Johnson, Haoshu Yang, Priya Chaudhary, Susan Bartolini, Norling Kho, Paul W. Noble, Sharon Isonaka, Peter Chen

**Affiliations:** ^1^Department of Medicine, Women’s Guild Lung Institute, Cedars-Sinai Medical Center, Los Angeles, California; ^2^Clinical Efficiency and Value, Cedars-Sinai Medical Center, Los Angeles, California; ^3^CDC COVID-19 Emergency Response Team.

In mid-December 2021, the B.1.1.529 (Omicron) variant of SARS-CoV-2, the virus that causes COVID-19, surpassed the B.1.617.2 (Delta) variant as the predominant strain in California.^§^ Initial reports suggest that the Omicron variant is more transmissible and resistant to vaccine neutralization but causes less severe illness compared with previous variants (*1*–*3*). To describe characteristics of patients hospitalized with SARS-CoV-2 infection during periods of Delta and Omicron predominance, clinical characteristics and outcomes were retrospectively abstracted from the electronic health records (EHRs) of adults aged ≥18 years with positive reverse transcription–polymerase chain reaction (RT-PCR) SARS-CoV-2 test results admitted to one academic hospital in Los Angeles, California, during July 15–September 23, 2021 (Delta predominant period, 339 patients) and December 21, 2021–January 27, 2022 (Omicron predominant period, 737 patients). Compared with patients during the period of Delta predominance, a higher proportion of adults admitted during Omicron predominance had received the final dose in a primary COVID-19 vaccination series (were fully vaccinated) (39.6% versus 25.1%), and fewer received COVID-19–directed therapies. Although fewer required intensive care unit (ICU) admission and invasive mechanical ventilation (IMV), and fewer died while hospitalized during Omicron predominance, there were no significant differences in ICU admission or IMV when stratified by vaccination status. Fewer fully vaccinated Omicron-period patients died while hospitalized (3.4%), compared with Delta-period patients (10.6%). Among Omicron-period patients, vaccination was associated with lower likelihood of ICU admission, and among adults aged ≥65 years, lower likelihood of death while hospitalized. Likelihood of ICU admission and death were lowest among adults who had received a booster dose. Among the first 131 Omicron-period hospitalizations, 19.8% of patients were clinically assessed as admitted for non–COVID-19 conditions. Compared with adults considered likely to have been admitted because of COVID-19, these patients were younger (median age = 38 versus 67 years) and more likely to have received at least one dose of a COVID-19 vaccine (84.6% versus 61.0%). Although 20% of SARS-CoV-2–associated hospitalizations during the period of Omicron predominance might be driven by non–COVID-19 conditions, large numbers of hospitalizations place a strain on health systems. Vaccination, including a booster dose for those who are fully vaccinated, remains critical to minimizing risk for severe health outcomes among adults with SARS-CoV-2 infection.

Periods of Delta and Omicron predominance (July 15–September 23, 2021, and December 21, 2021–January 27, 2022, respectively) were defined to correspond to peaks in SARS-CoV-2 hospitalizations during which each variant accounted for ≥50% of sequenced SARS-CoV-2 isolates in California (Supplementary Figure, https://stacks.cdc.gov/view/cdc/113987). RT-PCR–positive test results were determined via the hospital's internal flagging system for SARS-CoV-2 admissions, which incorporated laboratory results and provider documentation.^¶^ Vaccination status was ascertained through electronic linkage from the EHR to the California Immunization Registry (CAIR).** Patient demographic and clinical characteristics were abstracted from the EHR. For early Omicron-period hospitalizations (December 21–January 2), detailed chart review was performed by one of four clinicians to determine whether the reason for admission was likely or not likely due to COVID-19, following prespecified criteria.^††^

Patient demographic and clinical characteristics were compared between Delta- and Omicron-period hospitalizations, overall and stratified by vaccination status (partially vaccinated persons were excluded from stratified analyses because of small sample size). Because booster doses were not yet recommended during the period of Delta predominance,^§§^ Omicron-period patients who had received a booster dose were excluded from Delta- and Omicron-period comparisons of illness severity indicators (ICU admission, IMV, and death while hospitalized) among fully vaccinated persons. During Delta predominance, the EHR linkage to CAIR did not record booster doses. Fully vaccinated persons hospitalized during Delta predominance were assumed not to have received a booster dose. Among Omicron-period hospitalizations, these severity indicators were compared by four-level vaccination status (unvaccinated, partially vaccinated, fully vaccinated without a booster dose, and fully vaccinated with a booster dose). Patients who remained hospitalized as of January 27, 2022, were excluded from comparisons of death while hospitalized. Demographic and clinical characteristics were also compared between hospitalizations attributed to COVID-19 and those attributed to non-COVID-19 conditions during the early Omicron predominance period. Fisher’s exact tests were used to compare categorical variables and the Mann-Whitney U test was used to compare ordinal or continuous variables. Two-tailed p-values <0.05 were considered statistically significant. All analyses were conducted with R software (version 4.1.2; R Foundation). This study was reviewed and approved by the Cedars-Sinai Institutional Review Board.^¶¶^

Compared with 339 adults hospitalized during the Delta predominant period, the 737 adults hospitalized during the Omicron period included more fully vaccinated persons (39.6% versus 25.1%; p<0.01), and fewer unvaccinated persons (56.4% versus 71.1%; p<0.01) (Table 1). The median age increased both overall and among unvaccinated persons (Omicron = 64 years; Delta = 54 years; p<0.01), but not among fully vaccinated persons. The proportion of fully vaccinated adults who were Hispanic was higher during Omicron predominance (21.9%) than during Delta predominance (10.6%) (p = 0.02). Conversely, non-Hispanic White persons accounted for fewer admissions among fully vaccinated adults during Omicron predominance than during Delta predominance (46.6% versus 62.4%; p = 0.01). Fewer patients admitted during Omicron predominance than during Delta predominance received COVID-19–directed therapies, both among unvaccinated (57.9% and 81.7%; respectively) (p<0.01) and fully vaccinated adults (52.4% and 76.5%, respectively) (p<0.01). Compared with Delta-period patients, fewer Omicron-period patients required ICU admission (16.8% versus 23.3%; p = 0.01) or IMV (9.2% versus 13.6%; p = 0.03), and fewer died while hospitalized (4.0% versus 8.3%; p = 0.01). When stratified by vaccination status, however, differences in ICU admission and IMV between the two periods were not significant, despite lower percentages during Omicron predominance. Fewer fully vaccinated adults hospitalized during Omicron predominance died while hospitalized (3.4%) compared with those hospitalized during Delta predominance (10.6%) (p = 0.02). Among adults hospitalized during Omicron predominance, increasing vaccination was associated with lower likelihood of ICU admission (p = 0.02) and, among adults aged ≥65 years, lower likelihood of death while hospitalized (p = 0.04) (Figure). Fully vaccinated patients who had received a booster dose had the lowest likelihood of these outcomes.

**TABLE 1:** Demographic characteristics, clinical characteristics, and clinical outcomes among 1,076 hospitalized adults with SARS-CoV-2 infection by vaccination status and period of variant predominance — one hospital, California, July 15–September 23, 2021 (Delta period) and December 21, 2021–January 27, 2022 (Omicron period)

Characteristic	No. (%)								
	Total hospitalizations (N = 1,076)			Unvaccinated (n = 657)			Fully vaccinated (n = 377)		
	Delta period	Omicron period	p-value	Delta period	Omicron period	p-value	Delta period	Omicron period	p-value
**Total**	339	737	—	241	416	—	85	292	—
Vaccination status*^,†^									
Unvaccinated	241 (71.1)	416 (56.4)	<0.01	241 (100)	416 (100)	—	—	—	—
At least 1 dose	98 (28.9)	321 (43.6)	<0.01	—	—	—	85 (100)	292 (100)	—
Fully vaccinated	85 (25.1)	292 (39.6)	<0.01	—	—	—	85 (100)	292 (100)	—
Fully vaccinated and booster dose	—^§^	70 (9.5)	—	—	—	—	—^§^	70 (24.0)	—
**Age, yrs, median (IQR)**	60 (43–73)	66 (49–79)	<0.01	54 (38–68)	64 (48–78)	<0.01	71 (5–82)	69 (51–80)	0.36
**Sex**									
Men	190 (56.0)	377 (51.2)	0.15	130 (53.9)	221 (53.1)	0.87	52 (61.2)	142 (48.6)	0.05
Women	149 (44.0)	360 (48.8)		111 (46.1)	195 (46.9)		33 (38.8)	150 (51.4)	
**Race and ethnicity**									
White, non-Hispanic	163 (48.1)	336 (45.6)	0.47	105 (43.6)	184 (44.2)	0.94	53 (62.4)	136 (46.6)	0.01
Black, non-Hispanic	69 (20.4)	145 (19.7)	0.81	54 (22.4)	87 (20.9)	0.69	12 (14.1)	52 (17.8)	0.51
Hispanic	56 (16.5)	157 (21.3)	0.07	45 (18.7)	87 (20.9)	0.54	9 (10.6)	64 (21.9)	0.02
Asian, non-Hispanic	10 (2.9)	33 (4.5)	0.31	4 (1.7)	17 (4.1)	0.11	6 (7.1)	16 (5.5)	0.60
Other, non-Hispanic^¶^	41 (12.1)	66 (9.0)	0.12	33 (13.7)	41 (9.9)	0.16	5 (5.9)	24 (8.2)	0.64
**COVID-19 therapies received**									
Any	273 (80.5)	412 (55.9)	<0.01	197 (81.7)	241 (57.9)	<0.01	65 (76.5)	153 (52.4)	<0.01
Dexamethasone	245 (72.3)	360 (48.8)	<0.01	178 (73.9)	216 (51.9)	<0.01	57 (67.1)	129 (44.2)	<0.01
Remdesivir	234 (69.0)	293 (39.8)	<0.01	170 (70.5)	173 (41.6)	<0.01	54 (63.5)	106 (36.3)	<0.01
Other therapies**	76 (22.4)	92 (12.5)	<0.01	47 (19.5)	58 (13.9)	0.08	23 (27.1)	30 (10.3)	<0.01
**Intensive care unit admission**	79 (23.3)	124 (16.8)	0.01	55 (22.8)	79 (19.0)	0.27	20 (23.5)	34 (15.3)^††^	0.10
**Invasive mechanical ventilation**	46 (13.6)	68 (9.2)	0.03	37 (15.4)	45 (10.8)	0.11	8 (9.4)	19 (8.6)^††^	0.82
**Death while hospitalized**	28 (8.3)	22 (4.0)^§§^	0.01	19 (7.9)	14 (4.9)^¶¶^	0.21	9 (10.6)	6 (3.4)***	0.02

* Vaccination status was ascertained from the California Immunization Registry. Fully vaccinated adults were those who were not immunocompromised and had received the second dose of a 2-dose COVID-19 vaccine series or a single dose of a 1-dose product ≥14 days before receiving a positive SARS-CoV-2 test result associated with their hospitalization. Immunocompromised adults were considered fully vaccinated if they had received a third dose of a 3-dose primary series or a single dose of a 1-dose product ≥14 days before receiving a positive SARS-CoV-2 test result associated with their hospitalization. Fully vaccinated adults were considered to have received a booster dose if they had received an additional dose (third or fourth) of an mRNA COVID-19 vaccine ≥14 days before receiving a positive SARS-CoV-2 test result associated with their hospitalization. Adults whose positive SARS-CoV-2 test date was ≥14 days after the first dose of a 2-dose series (or second dose of a 3-dose series) but <14 days after receipt of the second dose (or third dose) were considered partially vaccinated, as were those who had received only a single dose of a 2-dose product (or 1 or 2 doses of a 3-dose series). Adults with no documented receipt of any COVID-19 vaccine dose before the test date were considered unvaccinated.

^†^ Partially vaccinated adults were not included in analyses stratified by vaccination status because of small sample size. However, they were included in overall proportions and comparisons not stratified by vaccination status; thus, the total number of patients exceeds the sum of fully vaccinated and unvaccinated patients.

^§^ Vaccination status was ascertained from the California Immunization Registry. Booster status was unavailable for hospitalizations before December 1, 2021.

^¶^ Includes Native Hawaiian, other Pacific Islander, American Indian, and Alaska Native persons, and persons of unknown race or ethnicity.

** Includes baricitinib, casirivimab-imdevimab, convalescent plasma, sotrovimab, and tocilizumab.

^††^ Denominator excludes 70 fully vaccinated patients who also received a booster dose.

^§§^ Denominator excludes 188 patients who remained hospitalized as of January 27, 2022.

^¶¶^ Denominator excludes 129 patients who remained hospitalized as of January 27, 2022

*** Denominator excludes 70 fully vaccinated patients who also received a booster dose and 43 patients who remained hospitalized as of January 27, 2022.

**FIGURE:**
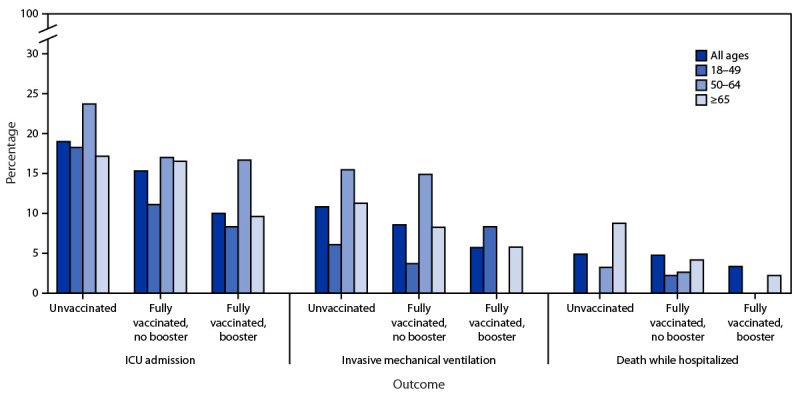
Intensive care unit admission, use of invasive mechanical ventilation, and death while hospitalized among 737 adults hospitalized with SARS-CoV-2 infection during Omicron variant predominance, by age group and vaccination status*^,^^†^ — one hospital, California, December 21, 2021– January 27, 2022 **Abbreviation:** ICU = intensive care unit. * The following were statistically significantly associated with increasing vaccination: ICU admission (all ages); death while hospitalized (age ≥65 years). ^†^ Percentages among partially vaccinated adults were included in analysis but are not displayed because of small sample size.

Of 131 early Omicron-period hospitalizations (December 21–January 2), 105 (80.2%) patients were assessed to have been likely admitted for COVID-19, and 26 (19.8%) were admitted primarily for non–COVID-19 conditions (Table 2). Compared with adults hospitalized for COVID-19, those hospitalized for other conditions were younger (median age 38 versus 67 years; p<0.01), more likely to have received at least one dose of a COVID-19 vaccine (84.6% versus 61.0%; p = 0.02), less likely to experience symptoms and signs of a COVID-like illness, and less likely to receive COVID-19–directed therapies. Among the 105 patients hospitalized for COVID-19, 63.8% had lower respiratory tract symptoms, 51.4% had abnormal chest radiography, and 39.0% had hypoxemia.

**TABLE 2:** Demographic and clinical characteristics and clinical outcomes among 131 adults hospitalized with SARS-CoV-2 infection during early Omicron variant predominance, by primary reason for admission — one hospital, California, December 21, 2021–January 2, 2022

Characteristic	No. (column %)			p-value
	Total hospitalizations (N = 131)	Hospitalizations not likely due to COVID-19 (n = 26)	Hospitalizations likely due to COVID-19 (n = 105)	
**Age, yrs, median (IQR)**	63 (38–79)	38 (29–62)	67 (47–79)	<0.01
**Sex**				
Men	61 (46.6)	11 (42.3)	50 (47.6)	
Women	70 (53.4)	15 (57.7)	55 (52.4)	0.67
**Race and ethnicity**				
White, non-Hispanic	59 (45.0)	9 (34.6)	50 (47.6)	0.28
Hispanic	32 (24.4)	10 (38.5)	22 (21.0)	0.08
Black, non-Hispanic	26 (19.8)	5 (19.2)	21 (20.0)	>0.99
Asian, non-Hispanic	5 (3.8)	2 (7.7)	3 (2.9)	0.26
Other, non-Hispanic*	9 (6.9)	0 (—)	9 (8.6)	0.20
**Vaccination status^†^**				
Unvaccinated	45 (34.4)	4 (15.4)	41 (39.0)	0.02
At least 1 dose	86 (65.6)	22 (84.6)	64 (61.0)	0.02
Fully vaccinated	80 (61.1)	20 (76.9)	60 (57.1)	0.07
Fully vaccinated and booster dose	18 (13.7)	4 (15.4)	14 (13.3)	0.76
**Initial symptoms and signs**				
Lower respiratory symptoms^§^	68 (51.9)	1 (3.8)	67 (63.8)	<0.01
Abnormal chest radiograph^¶^	55 (42.0)	1 (3.8)	54 (51.4)	<0.01
Hypoxemia	41 (31.3)	0 (—)	41 (39.0)	<0.01
Fever**	39 (29.8)	5 (19.2)	34 (32.4)	0.24
Gastrointestinal symptoms^††^	32 (24.4)	7 (26.9)	25 (23.8)	0.80
**Underlying medical conditions**				
Obesity (BMI ≥30)	46 (35.1)	8 (30.8)	38 (36.2)	0.65
Renal disease	14 (10.7)	2 (7.7)	12 (11.4)	0.74
Hypertension	13 (9.9)	0 (—)	13 (12.4)	0.07
Cardiovascular disease^§§^	11 (8.4)	0 (—)	11 (10.5)	0.11
Diabetes mellitus	6 (4.6)	0 (—)	6 (5.7)	0.60
Chronic pulmonary disease^¶¶^	2 (1.5)	0 (—)	2 (1.9)	>0.99
**COVID-19 therapies administered**				
Any	73 (55.7)	4 (15.4)	69 (65.7)	<0.01
Dexamethasone	63 (48.1)	3 (11.5)***	60 (57.1)	<0.01
Remdesivir	51 (38.9)	2 (7.7)^†††^	49 (46.7)	<0.01
Other therapies^§§§^	29 (19.8)	0 (—)	26 (24.8)	<0.01
**Intensive care unit admission**	17 (13.0)	2 (7.7)	15 (14.3)	0.52
**Invasive mechanical ventilation**	12 (9.2)	2 (7.7)	10 (9.5)	>0.99
**Death while hospitalized**	2 (1.9)^¶¶¶^	0 (—)****	2 (2.4)^††††^	>0.99

**Abbreviation:** BMI = body mass index.

* Includes Native Hawaiian, other Pacific Islander, American Indian, and Alaska Native persons, and persons of unknown race or ethnicity.

^†^ Fully vaccinated adults were those who were not immunocompromised and had received the second dose of a 2-dose COVID-19 vaccine series or a single dose of a 1-dose product ≥14 days before receiving a positive SARS-CoV-2 test result associated with their hospitalization. Immunocompromised adults were considered fully vaccinated if they had received a third dose of a 3-dose primary series or a single dose of a 1-dose product ≥14 days before receiving a positive SARS-CoV-2 test result associated with their hospitalization. Fully vaccinated adults were considered to have received a booster dose if they had received an additional (third or fourth) dose of an mRNA COVID-19 vaccine ≥14 days before receiving a positive SARS-CoV-2 test result associated with their hospitalization. Adults whose positive SARS-CoV-2 test date was ≥14 days after the first dose of a 2-dose series (or second dose of a 3-dose series) but <14 days after receipt of the second dose (or third dose) were considered partially vaccinated, as were those who had received only a single dose of a 2-dose product (or 1 or 2 doses of a 3-dose series). Adults with no documented receipt of any COVID-19 vaccine dose before the test date were considered unvaccinated.

^§^ Includes dyspnea, cough, and wheezing.

^¶^ Includes presence of opacities or nonspecific densities.

**Either documented temperature >100.4°F (38°C) on admission or identification of fever in a clinical note by the emergency physician or admitting provider.

^††^ Includes nausea, vomiting, and diarrhea.

^§§^ Includes coronary artery disease, congestive heart failure, arrhythmias, valvular heart disease, stroke, and peripheral vascular disease.

^¶¶^ Includes chronic obstructive pulmonary disease, pulmonary fibrosis, and asthma.

*** Dexamethasone was administered for neurosurgical indications (two) and for suspected bacterial meningitis (one).

^†††^ Remdesivir was administered in the setting of difficulty extubating after a gastrointestinal procedure (one) and for unclear indication in a patient admitted for psychiatric care (one).

^§§§^ Includes baricitinib, casirivimab-imdevimab, convalescent plasma, sotrovimab, and tocilizumab.

^¶¶¶^ Denominator does not include 25 patients who remained hospitalized as of January 11, 2022.

**** Denominator does not include two patients who remained hospitalized as of January 11, 2022.

^††††^ Denominator does not include 23 patients who remained hospitalized as of January 11, 2022.

## Discussion

Among adults hospitalized with SARS-CoV-2 infection at a single hospital in California during the Omicron-predominant period (December 21, 2021–January 27, 2022), COVID-19 vaccination, particularly receipt of a booster dose, was associated with lower likelihood of ICU admission, and, among adults aged ≥65 years, lower likelihood of death while hospitalized. Compared with the period of Delta predominance, a higher proportion of adults hospitalized during Omicron predominance were fully vaccinated. Consistent with earlier findings (*3*), Omicron-period hospitalizations were associated with a lower likelihood of ICU admission, IMV, and death while hospitalized, compared with Delta-period hospitalizations. However, the proportion requiring ICU admission and IMV did not differ significantly when stratified by vaccination status, suggesting that much of the lower disease severity observed during Omicron predominance might be driven by increased population-level vaccine-conferred immunity. These findings support the continued importance of COVID-19 vaccination, including booster doses, in mitigating the risk of severe illness associated with SARS-CoV-2 infection.

From mid-July through mid-December 2021, the proportion of fully vaccinated adults in Los Angeles County increased nearly 20%, from approximately 65% to 77%,*** but the proportion of SARS-CoV-2 hospitalizations occurring in fully vaccinated adults increased almost 60%, from approximately 25% to 40%. The increase in the percentage of fully vaccinated Hispanic adults and the decrease in the percentage of non-Hispanic White adults hospitalized between the two periods likely reflect increased vaccination coverage among Hispanic persons during fall 2021. Increases in infections among vaccinated persons during the period of Omicron predominance were likely driven both by waning vaccine-derived immunity over time and by relative resistance to vaccine neutralization in the Omicron variant compared with the Delta variant (*2*,*4*). This is consistent with the observed decline in effectiveness of 2-dose vaccination against COVID-19 hospitalization during the Omicron period (*5*). A previous study also found that, compared with the period of Delta predominance, the period of Omicron predominance in Los Angeles County was associated with a decrease in the degree of protection against COVID-19 and hospitalization (*6*). Despite this, COVID-19 vaccination, including a booster dose, was associated with lower likelihood of ICU admission during the Omicron period, and lower likelihood of death among adults aged ≥65 years, who are at higher risk for severe outcomes when hospitalized with COVID-19 (*7,**8*).

Early reports suggest that the Omicron variant has lower replication competence in lung parenchyma,^†††^^,^^§§§^ possibly contributing to a decreased severity of illness compared with earlier variants (*3*). However, among patients hospitalized for COVID-19 during the early Omicron predominant period, most had lower respiratory symptoms and abnormal chest imaging, approximately one third had hypoxemia, and 10% required IMV. These findings demonstrate that, despite observed changes compared with Delta, Omicron variant infection still causes severe lower respiratory illness. Similar data on patient symptoms were not available for Delta-period hospitalizations. However, fewer Omicron-period patients received COVID-19-directed therapies, which might suggest lower proportion with hypoxemia, compared with Delta-period patients. Alternatively, this change might have been driven by changes in prescribing practices or other unmeasured factors.

Approximately 20% of SARS-CoV-2 admissions during early Omicron predominance were likely for reasons other than COVID-19, a proportion even higher among young and vaccinated adults. Given high rates of SARS-CoV-2 community transmission, this is not unexpected. This estimate stands in contrast to an estimated 63% of patients admitted with incidental SARS-CoV-2 infection reported from South Africa (*9*). While this difference might be driven, in part, by differences in demographics and population immunity, the present study’s classification methodology might have overestimated the number of persons whose admission was driven by COVID-19. One third of patients classified as having been admitted for COVID-19 received no COVID-19–directed therapies. Alternatively, high population-level immunity from vaccination, previous SARS-CoV-2 infection, or both might have modulated the clinical presentation of patients with COVID-19 during Omicron predominance and atypical presentations might have been underrecognized (e.g., exacerbations of chronic medical conditions), or lesser illness severity might have resulted in fewer therapies. However, the pandemic health care burden is not limited to hospitalizations for symptomatic COVID-19. Even patients with positive SARS-CoV-2 test results admitted for non-COVID-19 conditions require isolation rooms and use of personal protective equipment and might transmit infection to health care workers, exacerbating staff shortages.

The findings in this report are subject to at least six limitations. First, sequencing data were not available to identify the SARS-CoV-2 variant. However, based on California genomic surveillance data, which is based on sequencing of ≥10% of all positive RT-PCR tests in the state,^¶¶¶^ and on recent genomic surveillance for Los Angeles County (*7*), the Delta and Omicron variants accounted for the majority of sequenced isolates throughout their respective predominance periods. Second, the proportion of Omicron-period hospitalizations attributed to COVID-19 could not be compared with earlier periods, so it is unclear whether the proportion represented a change from an earlier period. Third, the study might have been underpowered to detect Omicron-specific reductions in illness severity after stratifying by vaccination status. Fourth, the analysis could not account for the interval since the last dose of COVID-19 vaccine, which might have been longer among Omicron-period patients. Fifth, there might have been incomplete ascertainment of deaths in the recent weeks of Omicron predominance; severely ill patients might remain hospitalized and might be at high risk of death. A longer period of observation might have reduced differences in death between the two periods. Finally, these findings are from a single hospital in Los Angeles and cannot be generalized to the United States. However, the hospital has a large catchment area in a racially and ethnically diverse region.

In this single-hospital study, adults hospitalized with SARS-CoV-2 infection during Omicron predominance had less severe illness compared with adults hospitalized during Delta predominance. Much of this effect appears to be driven by increased proportion of patients who were fully vaccinated. Approximately 20% of Omicron-period hospitalizations among adults with a positive SARS-CoV-2 RT-PCR result were driven by non–COVID-19 conditions, which might be attributed to high SARS-CoV-2 community transmission and high population vaccination coverage. COVID-19 vaccination was associated with lower likelihood of ICU admission during Omicron predominance. COVID-19 vaccination, including a booster dose for those who are fully vaccinated, is critical to minimizing the risk for severe health outcomes among adults with COVID-19.

**Summary**

**What is already known about this topic?**

The SARS-CoV-2 Omicron variant became predominant in the United States in mid-December 2021, coinciding with a rise in SARS-CoV-2–associated hospitalizations.

**What is added by this report?**

Among adults hospitalized with SARS-CoV-2 infection during Omicron predominance, COVID-19 vaccination, including with a booster dose, was associated with lower likelihood of intensive care unit admission. Compared with patients during the period of Delta predominance, Omicron-period patients had less severe illness, largely driven by an increased proportion who were fully vaccinated. Approximately 20% of early Omicron-period hospitalizations were for non–COVID-19 conditions, particularly among young and vaccinated adults.

**What are the implications for public health practice?**

COVID-19 vaccination, particularly a booster dose, continues to be critical in mitigating the health care burden of the Omicron variant.

## Footnotes

* These authors contributed equally to this report.

† These senior authors contributed equally to this report.

§
https://covid19.ca.gov/variants/

¶ This flagging can be triggered in one of two ways: either by a laboratory report indicating a positive SARS–CoV-2 RT-PCR test result from any time during hospitalization or during a 14-day lookback window preceding admission, which included tests performed in ambulatory and inpatient settings, or by admitting physician confirmation of RT-PCR positivity from an outside facility via patient interview, during which time a patient was queried about positive RT-PCR test results and any related COVID-19 symptoms over the preceding 14 days. Hospitalizations were included in the study if they occurred among adults without another hospitalization associated with a positive SARS-CoV-2 RT-PCR result during the preceding 90 days, or if there was a hospitalization associated with a positive RT-PCR result during the preceding 90 days, but the patient’s symptoms had resolved before readmission as determined by the admitting provider.

** Fully vaccinated adults were those who were not immunocompromised and had received the second of a 2-dose COVID-19 vaccine series or a single dose of a 1-dose product ≥14 days before receiving a positive SARS-CoV-2 test result associated with their hospitalization. Immunocompromised adults were considered fully vaccinated if they had received a third dose of a 3-dose primary series or a single dose of a 1-dose product ≥14 days before receiving a positive SARS-CoV-2 test result associated with their hospitalization. Fully vaccinated adults were considered to have received a booster dose if they had received an additional dose (third or fourth) of an mRNA COVID-19 vaccine ≥14 days before receiving a positive SARS-CoV-2 test result associated with their hospitalization. Adults whose positive SARS-CoV-2 test date was ≥14 days after the first dose of a 2-dose series (or second dose of a 3-dose series) but <14 days after receipt of the second dose (or third dose) were considered partially vaccinated, as were those who had received only a single dose of a 2-dose series (or 1 or 2 doses of a 3-dose series). Adults with no documented receipt of any COVID-19 vaccine dose before the test date were considered unvaccinated.

†† Chart review included notes by the emergency department provider, admitting provider, initial infectious disease consultant (when consulted), and discharging provider when available. Admissions associated with a positive SARS-CoV-2 RT-PCR result were classified as likely due to COVID-19 if the admitting provider affirmed that COVID-19 was the reason for admission or, in the absence of explicit determination, if reviewers could not determine a clear alternative reason for admission that was not plausibly linked to SARS-CoV-2 infection. Alternative reasons for admission included uncomplicated labor, a surgical procedure, trauma, psychiatric care, or a medical diagnosis not plausibly linked to COVID-19 (cellulitis [six patients], gastrointestinal bleeding [two], small bowel obstruction [two], and osteomyelitis [one]). Exacerbations of chronic conditions (e.g., congestive heart failure, chronic obstructive pulmonary disease, and asthma) were attributed to COVID-19. Any positive RT-PCR test results ≥7 days after initial negative test results on admission were considered to represent nosocomial SARS-CoV-2 infections, and therefore, the admission was not attributed to COVID-19. This applied to three hospitalizations included in this report.

§§
https://www.cdc.gov/media/releases/2021/p0924-booster-recommendations-.html

¶¶ 45 C.F.R. part 46; 21 C.F.R. part 56.

***
https://covid19.ca.gov/vaccination-progress-data/

†††
https://www.researchsquare.com/article/rs-1189219/v1

§§§
https://www.researchsquare.com/article/rs-1211792/v1
